# Construction Notes

**Published:** 1894-04-07

**Authors:** 


### CONSTRUCTION NOTES.

**Hospital Extension at St Helen's, Liverpool.**

The erection of a new wing at the St. Helen's Cottage Hospital, which is supported by public subscription, and the penny-a-week system for working men, is now approaching completion, and increased accommodation for thirty-two beds will be provided. This extension joins the main building, and is approached by a corridor through the present entrance hall. A ward kitchen, nurses' duty-room, and small room for convalescents, are arranged so as to be near the administrative block. The other end of the ward has a bath-room, lavatory, and pantry on each floor. A smoking balcony for men, and a fire escape balcony and staircase is supplied. The whole of the floors are constructed on the fireproof system. The estimated cost of the building, furniture, &c., is about £4,000. Mr. James Gandy is the architect, the contract for whose designs was let to Mr. Peter Tickle, of Parr.

**Todmorden Infectious Diseases Hospital.**

Owing to the liberality of the Fielden family to the town of Todmorden, buildings and land, which comprise the new infectious diseases hospital, have been handed over to the Hospital Committee of the Todmorden Local Board on behalf of the Joint Hospital Committee of the Todmorden Union.

The hospital comprises four blocks of buildings. The administration block affords accommodation for the staff. Behind this is the principal hospital, in which are one ward for children with two beds, one for adults with eight beds, and one with six beds, in addition to the nurses' rooms, hall, &c. The isolation block will be used for cases of diphtheria and other highly infectious cases. In this are two wards with one bed each, and two wards with two beds each. The fourth block is set apart for laundry purposes and disinfecting clothing, &c. All the buildings are fitted up in the most approved style, from plans by Mr. John Sutcliffe, architect, Todmorden. The entire cost is about £6,000, and has been defrayed by Mr. J. A. Fielden, Centre Vale, Todmorden, in accordance with the wish of his deceased father, Mr. Samuel Fielden.

**New Infectious Hospital at Bolton.**

It has been considered for some time that a new building for the treatment of infectious cases was necessary, in order to relieve somewhat the present fever hospital, which it is intended now to use solely for surgical cases occurring in the workhouse, and the new building in course of erection, at an estimated cost of £2,800, will be wholly utilized for the treatment of all infectious cases) except small-pox and typhus, for which provision is made at Rumworth), and will contain eight beds. The hospital will consist of four wards for three beds each, and two nurses' rooms. All these rooms are to be connected only by an open verandah on the south side, and this verandah will be divided in the centre by a fence which will cut the entire plot in two, and so keep the two halves of the hospital separate. To the right at the back of the hospital is the administrative block, which will contain caretakers' rooms, bed-rooms for four nurses, bath-rooms, store-rooms, &c. The building to the left will consist of washhouse, disinfecting-room, mortuary, coal-place, &c. A thorough system of disinfecting apparatus is to be placed here. Messrs. Woodhouse and Potts, F.R.I.B.A., are the architects for the building, and the contract has been entrusted to Mr. S. J. Hodgkin, of Farnworth.

**New Hospital at Ilkeston.**

Lord Belper opened this new hospital at Ilkeston, which was erected at a cost of £3,000 on a site presented by Mr. E. N. Mundy, J.P., C.C. For many years the accommodation at Ilkeston has been too scant, and the need of extended premises has long been felt. These buildings are substantially built of red brick, the roofs being covered with brown Broseley tile. The medical and administrative departments face the main road leading from Ilkeston to Heanor, and are two storeys in height, containing, on the ground floor, entrance hall, nurses' room, kitchen, scullery, laundry, &c. The first floor contains matron's and nurses' bed-rooms, bath-room, and children's ward for eight beds. Covered corridors connect the administrative department with the special wards. The women's ward for six beds is constructed so as to correspond with the laundry wing of the administrative department. This ward is so arranged as to form an out-patients' department at a future period. The men's ward for twelve beds is constructed as a parallel building, with the administrative department at the extreme end of the main corridor, and has a nurses' wing attached. An operating-room is also provided at the end of the main covered corridor. A mortuary and other out-buildings are provided for. The interior of the wards is fitted up with all modern appliances, including electric bells and speaking tubes communicating with each ward and bath-room, and also with the administrative department. The total cost of the building is £3,000. The contractor for the brickwork, &c., of the building was Mr. W. E. Shaw, of Ilkeston. The whole of the works have been carried out under the personal supervision of the architect, Mr. C. W. Hunt, A.R.I.B.A., of Ilkeston, who has given his services gratuitously in the preparation of the plans, specifications, &c.

